# Block & replacement regimen is more cost effective than titration regimen in treating thyrotoxicosis in children, without more harm

**DOI:** 10.1186/1687-9856-2015-S1-P105

**Published:** 2015-04-28

**Authors:** MK Anne Kwok, PT Cheung

**Affiliations:** 1 Department of Pediatrics & Adolescent Medicine, Queen Mary Hospital, Hong Kong

## Aim

The first line treatment of thyrotoxicosis in our unit is anti-thyroid medication for 2 years – longer if unstable control. Since 2010, we changed the anti-thyroid treatment protocol from block and replacement (B&R) to titration regimen (TIT), after the Cochrane review showed that B&R was associated with similar remission rate but more frequent adverse effects than TIT. We aimed at comparing these 2 regimens in our pediatric patients.

## Methods

Patients who received TIT from 2010 to 2014 for relapse of thyrotoxicosis and previously had at least 1 full course of B&R were reviewed. Those who had less than 2 years of TIT, either because they received surgery or radioactive iodine within 2 years, or they have not yet finished the course, were excluded from analysis.

The course of B&R versus TIT was compared within each individual. For those who had previously multiple courses of B&R, the latest course was used for analysis.

Occurrence of adverse drug side effects, length of the anti-thyroid treatment course, number of clinic visits and thyroid function tests during the course, and frequency of abnormal free thyroxine levels were reviewed and analyzed by paired t-test.

## Results

27 patients, who received B&R previously, were put on TIT for relapse of thyrotoxicosis after 2010. 17 patients were excluded from the analysis as they were on TIT for less than 2 years, 9 of them had surgery or radioactive iodine within 2 years after starting TIT.

Among the 10 patients (4 males, 6 females, age 8-16 years) analysed, no significant drug side effect was experienced during both treatment periods. While on TIT, patients required longer duration of treatment (average 23% longer, p=0.005) with more clinic visits (average 50% increase, p=0.003) and thyroid function tests (average 85% increase, p=0.002).

They needed more frequent clinic visits (average 22% increase, p=0.04) and thyroid function tests (average 50% increase, p=0.007) per year. They had higher number of abnormal free thyroxine levels per year (average 28% more frequent, p=0.023). The percentage of abnormal free thyroxine levels dropped insignificantly by 2.6% (p=0.645).

The estimated drug cost for 2 year’s B&R was ~397 HKD (51.2 USD / 54.6 AUD), while 2 years’ TIT cost ~177 HKD (22.8 USD / 24.3 AUD).

## Conclusion

Both B&R and TIT are safe without significant adverse effect. B&R seems more cost effective with shorter treatment duration and less frequent clinical and biochemical monitoring, even drug cost is slightly higher.

